# Random Time‐Space Coding Metasurfaces for Spatial Control of the Temporal Statistics of Electromagnetic Fields

**DOI:** 10.1002/advs.202521637

**Published:** 2026-03-15

**Authors:** Jia Cheng Li, Jiang Han Bao, Che Liu, Tie Jun Cui

**Affiliations:** ^1^ State Key Laboratory of Millimeter Waves Southeast University Nanjing China

**Keywords:** random electromagnetic fields, random time‐space coding metasurfaces (RTCM), spatial control, temporal statistics

## Abstract

Conventional methods to control electromagnetic (EM) fields via digital coding metasurfaces are basically focused on generating deterministic EM responses, such as beam forming, holographic imaging, and harmonic modulation, by employing specific coding patterns or sequences. In contrast, controlling random EM fields aims to produce EM fields with target temporal statistical properties, such as spatial mean and variance distributions. Here, we propose a framework to control the spatial distribution of temporal statistical properties of EM fields via random time‐space coding metasurfaces (RTCM). We establish a statistical model of RTCM in probability space and reveal that the spatial mean and variance distributions of EM fields are determined by the marginal and pairwise joint distributions of the random codes. Time‐varying random EM fields with desired mean and variance distributions are generated by the sampling codes constrained by these distributions. We further show that simultaneously direct transmission and jamming can be achieved via the spatial mean and variance peaks, respectively. This work extends the metasurface paradigm into the probability domain, marking a shift from deterministic response design to probabilistic structuring, and paving the way for new approaches in communication, information security, and EM countermeasures.

## Graphical abstract

A probabilistic framework based on random time‐space coding metasurfaces enables control of the spatial distribution of electromagnetic fields temporal statistics. By tailoring the marginal and joint distributions of random codes, electromagnetic fields with desired mean and variance patterns are realized, enabling simultaneous transmission and jamming. This work marks a shift from deterministic to probabilistic electromagnetic field control.

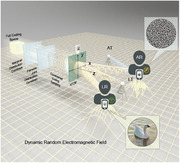

## 1 Introduction

Metasurfaces, composed of numerous subwavelength units with the capability to induce phase shift [1, 2, 3, 4], modulate amplitude [5, 6, 7], and convert polarization [8, 9, 10, 11], have emerged as a powerful platform for controlling electromagnetic (EM) fields. They enabled a wide range of functions, including anomalous reflection and refraction [12, 13, 14, 15], energy absorption [16, 17, 18, 19], and holographic imaging [20, 21, 22, 23]. With the introducing of digital coding and programmable technologies, time‐ space coding metasurfaces have been developed to further extend this capability into the time domain [24, 25, 26, 27], enabling applications such as harmonic manipulation and novel architecture transmitter [28, 29, 30, 31, 32, 33]. However, most existing metasurface studies are focused on deterministic control, where carefully designed coding patterns can generate specific EM responses. In such approaches, a predefined coding pattern or sequence will directly determine the resulting scattering profile or harmonic spectrum. This deterministic framework inherently excludes statistical variability and stochastic processes.

In contrast, randomness plays an essential role in practical systems, particularly in wireless communication and radar applications [34, 35, 36, 37]. In antenna array technologies, structural randomness is imposed on individual array elements through random thinning and controlled defects [38, 39, 40, 41]. Such structural randomness can improve the radiation characteristics, including the main‐lobe gain, beamwidth, and sidelobe suppression, while potentially reducing mutual coupling or system complexity. Nevertheless, in these approaches, the randomness primarily functions as a structural design strategy for shaping a static beam pattern, and the resulting EM field does not exhibit intrinsic temporal statistical evolution.

Beyond the traditional array technologies, metasurfaces have also demonstrated potential in manipulating the random EM fields, most notably by generating random scattering patterns through random phase or coding distributions. Such approaches provide advantages including broadband operation, wide‐angle coverage, multi‐polarization functionality, and reduction of scattering signatures [42, 43, 44, 45, 46, 47, 48, 49]. However, these implementations remain fundamentally static, as the fixed coding patterns produce time‐invariant random scattering patterns.

Recent advances have introduced the temporal variations into random coding schemes, enabling the metasurfaces to generate stochastic EM processes with Gaussian statistics and tailored spectral characteristics [50]. Other approaches have explored hybrid coding strategies that combine static focusing or beam‐steering patterns with time‐varying random sequences, thereby producing EM fields with partially deterministic and partially random characteristics, aiming to enhance the performance metrics such as coverage probability and communication security [51, 52]. Nevertheless, in these frameworks, the statistical structure of the EM field is not treated as an independently controllable physical quantity. Instead, the underlying mechanism remains rooted in omnidirectional random scattering induced by the unconstrained random coding patterns without prior statistical design, as predicted by the superposition principle. Such a principle limits the degrees of freedom achievable in the statistical control of EM fields, such as fields containing arbitrarily shaped regions of static and random behavior.

In this study, we propose a general statistical framework for controlling the spatial distributions of the temporal statistical properties of EM fields using RTCM. By formulating the EM response of RTCM in the probability space, we introduce the spatial mean and variance distributions as descriptors of static and random varying components of the EM fields. We show that the marginal and pairwise joint distributions of the dynamic random codes directly determine the spatial mean and variance of the resulting fields. Leveraging the random codes obtained via constrained sampling under specified probabilistic constraints, RTCM readily generates the EM fields exhibiting tailored spatial distributions of the temporal statistical properties. Both analytical and experimental results validate the theoretical framework. We demonstrate a proof‐of‐concept implementation where simultaneously direct transmission and jamming are realized via spatial statistical controls. This paradigm shift, from deterministic pattern design to probabilistic structuring of spatial statistical properties, paves the way for new approaches in metasurfaces research and its applications in communication, information security, and electronic countermeasures.

## 2 Theory

### 2.1 Principles for Spatial Control of the EM field Temporal Statistics

**FIGURE 1:**
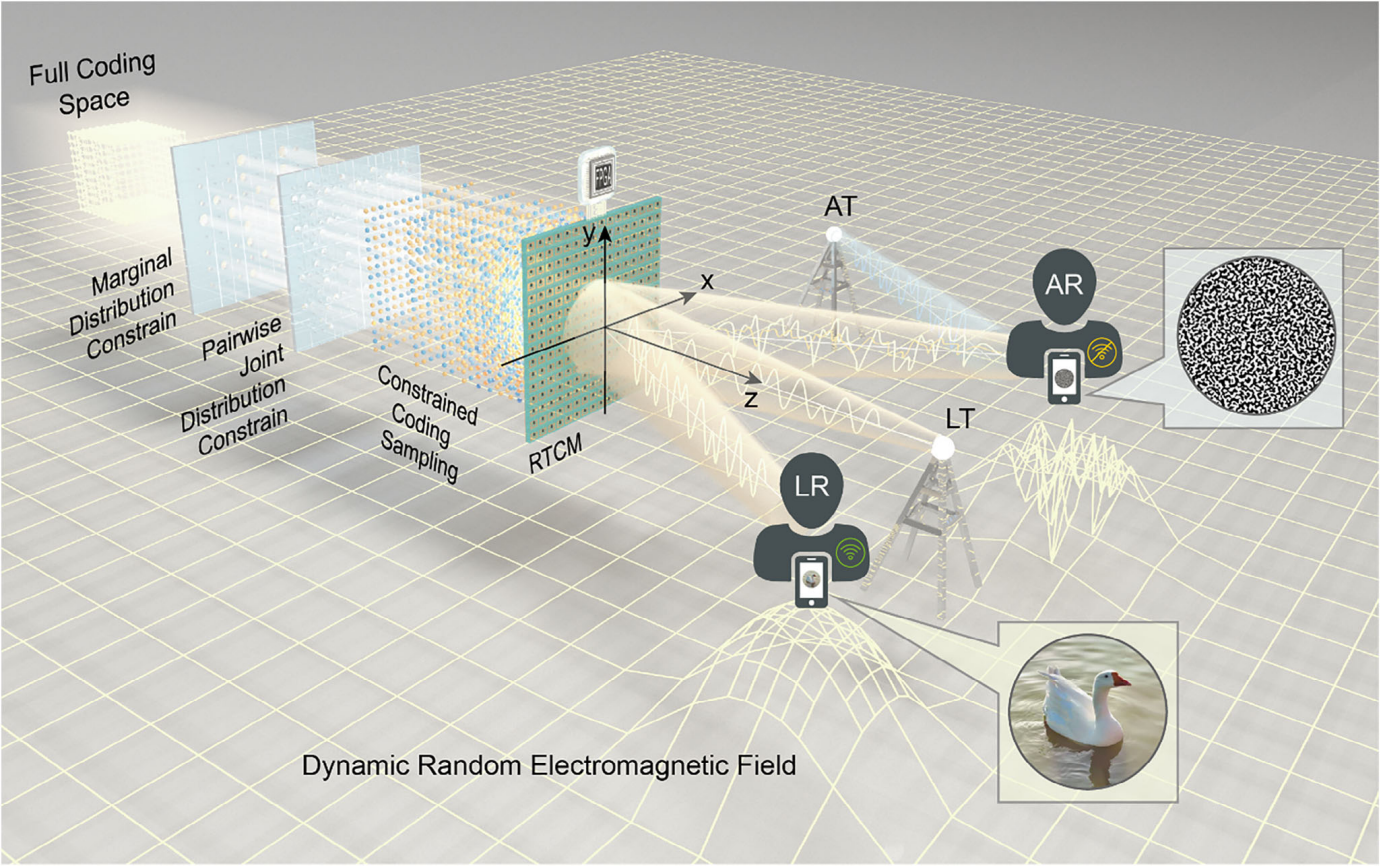
Conceptual schematic of controlling the spatial distribution of EM field temporal statistics via RTCM. By tailoring the statistical properties of scattered fields, RTCM preserves reliable transmission along a legitimate link from the legitimate transmitter (LT) to the legitimate receiver (LR), while intentionally degrades adversarial link from the adversarial transmitter (AT) to the adversarial receiver (AR).

Figure 1 illustrates the principle to control the spatial distributions of the temporal statistical properties of the EM field using RTCM, where the metasurface generates a dynamic random EM field containing locally time‐invariant static regions and time‐varying random noise regions. For a 1‐bit coding metasurface of size *M*×*N*, all possible coding patterns constitute the full coding space {0,1}*^MN^*. To generate the EM fields with a prescribed mean distribution, a marginal distribution constraint is imposed on this full coding space, yielding a constrained subspace. Subsequently, a pairwise joint distribution constraint is applied to further refine the subspace so that the variance distribution of EM fields is satisfied. Finally, random codes are sampled from this constrained subspace and drive RTCM, which produces dynamic random EM scattering fields with tailored spatial distributions of temporal statistical properties. In this statistical structure of EM fields, a receiver located in regions of large mean and small variance perceives deterministic signals, whereas a receiver in regions of large variance and small mean experiences strong jamming.

The model is now developed in greater detail. Consider a single tone wave with frequency *ω*_0_ and amplitude *A*_0_ transmitted from a radio source. The received signal can be expressed as *r*(*t*) = (μ + σ*n*(*t*))exp (*j*ω_0_ *t*) where *μ* represents the static time‐invariant component, *n*(*t*) denotes a normalized random noise component, and *σ* is the standard deviation of the random noise. By tuning the relative weights of *μ* and *σ*, the received signal can be steered between a static time‐invariant state and a random noise‐dominated state. Moreover, since the received signal is proportional to the EM field, the parameters *μ* and *σ* are directly linked to the spatial mean and variance distributions of the scattering EM fields. The scattering field generated by a 1‐bit coding metasurface is modeled as (see Note S1 for details)

(1) $$\left\{\begin{array}{c} f_{r}\left(t|r_{t},r_{r}\right)=A_{0}\exp\left(j\omega_{0}t\right)L\left(r_{t},r_{r}\right)f_{c}\left(t|r_{t},r_{r}\right)\\ f_{c}\left(t|r_{t},r_{r}\right)=\sum_{l=1}^{\mathrm{MN}}{\left(-1\right)}^{s\left(t|l\right)}\exp\left(j\Phi_{\mathrm{path}}\left(l\right)\right) \end{array}\right.$$

where *L*(**r**_t_,**r**_r_) = *C*_0_ *F*_u_(**r**_r_)/(*R*_r2ms_ *R*_ms2t_)^2^ denotes the loss coefficient from transmitter to receiver, *C*_0_ is the amplitude coefficient, *F*_u_(**r**_r_) refers to the normalized radiation pattern of a single unit, *R*_r2ms_ and *R*_ms2t_ are the distance from the metasurface center to the receiver, and from transmitter to the metasurface center, respectively. *f*_c_(*t*|**r**_t_,**r**_r_) denotes the time‐space coding factor (TSCF). Term of (−1)*^s^* ^(^ *^t^* ^|^ *^l^* ^)^ represents the 1‐bit reflection response of the *l‐*th unit located at *m*‐th row and *n*‐th column, and *s*(*t*|*l*)∈{0,1} is the corresponding state function. Φ_path_(*l*) = ‐*k*_0_(*R*_r2u_(*l*)+*R*_u2t_(*l*)) is the phase accumulation along the propagation path, *k*_0_ is the wave vector in free space, *R*_u2t_ and *R*_r2u_ denote the distances from the transmitter to the unit and from the unit to the receiver, respectively.

Equation (1) indicates that the random scattering EM fields are directly proportional to the TSCF, whose temporal variation originates from the time‐varying coding patterns. And thus, the far‐field scattering is governed by the cumulative contribution of numerous randomized element responses *s*(*t*|*l*), giving rise to its statistical properties. Consequently, tailoring the temporal statistical properties of the TSCF provides a direct means to adjust the spatial distribution of the temporal statistical properties of the scattering EM fields.

The temporal mean and variance of a random signal characterize its average value and the degree of fluctuation around it. For TSCF, they are defined as $\mu_{\mathrm{fc}}\left(r_{t},r_{r}\right)=E\left(f_{c}\left(t|r_{t},r_{r}\right)\right)\;\mathrm{and}\sigma_{\mathrm{fc}}^{2}\left(r_{t},r_{r}\right)=E\left(\left(f_{c}\left(t|r_{t},r_{r}\right)\right)-\mu_{\mathrm{fc}}\left(r_{t},r_{r}\right)\right)\times (f_{c}\left(t|r_{t},r_{r}\right)-\mu {\left(r_{t},r_{r})\right)}^{*}$, respectively, where (∙)^*^ denotes the adjoint operator, and E(·) denotes the mean operator. To unify the dimensions, we define *P_μ_* = *μ_fc_* (*μ_fc_*)^*^ as the power of the mean component, and *P_σ_* = (*σ_fc_*)^2^ as the power of the random fluctuating component.

Each unit of the RTCM can be set to either 0 or 1, with probabilities *p*_1_(*l*) = Pr(*s*(*l*) = 1) and *p*_0_(*l*) = Pr(*s*(*l*) = 0), respectively, such that *p*_0_(*l*)+*p*_1_(*l*) = 1. The mean power *P_μ_* could be driven as (see Note S2 for details)

(2) $$P_{\mu }={\left|\left(2{\left(p_{1}\right)}^{T}-1^{T}\right)\exp\left(j\Phi_{\mathrm{path}}\right)\right|}^{2}$$

where **p**_1_ = [*p*_1_(1),*p*_1_(2),…,*p*_1_(*MN*)]^T^, **Φ**_path_ = [Φ_path_(1),Φ_path_(2),…, Φ_path_(*MN*)]^T^, and **1** = [1,1,…,1]^T^ denotes the vector of ones. [∙]^T^ denotes the transpose operator. In addition, the phase component, defined as Φ*_μ_* = Arg((2(**p**_1_)^T^‐**1** ^T^)exp(*j* **Φ**_path_)), characterizes the mean phase distribution of the scattering field. Since Φ_path_(*l*) is a function of the observation angles, the mean power varies spatially. As indicated by Equation (2), the spatial distribution of mean power can be fully controlled by tailoring the marginal probabilities **p**_1_, enabling the realization of arbitrary mean power patterns. Specifically, when all units have equal likelihood of being 0 or 1 (*p*_0_(*l*) = *p*_1_(*l*) = 0.5), mean power is uniformly zero [46].

On the other hand, the fluctuating power *P_σ_* is closely related to the pairwise joint distribution *p_ab_* (*l*,*o*), which describes the likelihood that the *l*‐th and *o*‐th units assume states *a* and *b*, respectively, and could be driven as (see Note S2 for details)

(3) $$P_{\sigma }=4{\langle P,C\rangle }_{F}$$

where **P** = **P**_11_−**p**_1_(**p**_1_)^T^, **C** = cos(**Φ**_path_ **1** ^T^−**1**(**Φ**_path_)^T^), 〈∙,∗〉_F_ is the Frobenius inner product. **P**_11_ = [*p*_11_(*l*,*o*)] denotes the pairwise joint probability matrix. Equation (3) indicates that, through proper design of the marginal probability distribution **p**_1_ and the pairwise joint probability distribution **P**_11_, the fluctuating power can be tailored to realize any desired spatial distribution.

Consequently, to ensure that the constructed **P**_11_ is valid, it should satisfy the following probabilistic constraints (see Note S2 for details): (1) the general constraint that 0≤*p*_ab_(*l*,*o*)≤1; (2) the uniform condition that **p**_00_+**p**_01_+**p**_10_+**p**_11_ = **11** ^T^ (3) the dialog conditions that *diag*(**P**_01/10_) = **0** and *diag*(**P**_00/11_) = **p**_0/1_; (4) the symmetry conditions that **P**_01_ = (**P**_10_)^T^ and **P**_00/11_ = (**P**_00/11_)^T^; (5) the consistency conditions that **P**_00_+**P**_01_ = **p**_0_ **1** ^T^ and **P**_10_+**P**_11_ = **p**_1_ **1** ^T^; (6) the dependent conditions that **P**_00_ = **11** ^T^‐**p**_1_ **1** ^T^‐**1**(**p**_1_)^T^+**P**_11_ and **P**_01_ = (**P**_10_)^T^ = **1**(**p**_1_)^T^‐**P**_11_; (7) the boundary condition that max{0,*p*_1_(*l*)+*p*_1_(*o*)‐1}≤*p*_11_(*l*,*o*)≤min{*p*_1_(*l*),*p*_1_(*o*)}; (8) The positive semi‐definite condition of **P**⪰**0**.

The dialog conditions indicate that the marginal distribution information is embedded in the pairwise joint probabilities. The dependent conditions further imply that **P**_11_ can be used to characterize the other pairwise joint probabilities, since only one of **P**_00_, **P**_01_, **P**_10_, and **P**_11_ is independent. In this way, by carefully designing both the marginal distribution **p**_1_ and the pairwise joint probability **P**_11_, one can realize the desired mean and fluctuating power distributions of TSCF, thereby controlling the spatial distribution of the temporal statistical properties of the scattering EM field.

### 2.2 Simulation of Spatial Control of the EM field Temporal Statistics

To produce EM fields with prescribed mean and fluctuating power distributions, the marginal distribution **p**_1_ is first constructed from the target mean power distribution, since mean power depends solely on **p**_1_. Subsequently, the pairwise joint probability distribution **P**_11_ is constructed under the constraint of the obtained **p**_1_ and in accordance with the target fluctuating power distribution. Once both **p**_1_ and **P**_11_ are obtained, random codes can be sampled within the constrained probabilistic subspace. Finally, driven by these sampling random codes, the RTCM achieves the desired spatial control of the temporal statistical properties of EM fields. Moreover, since the random time‐space codes are generated under constraints of the marginal and pairwise joint probability distributions, which are time‐invariant, the random EM process can be regarded as ergodic.

Obtaining an exact solution for **p**_1_ and **P**_11_ that precisely generates the required mean and noise distributions is extremely challenging due to the high dimensionality of the feasible solution space. Therefore, a more practical approach is to find the optimal **p**_1_ and **P**_11_ that generate the mean and fluctuating power distributions as close as possible to the targets by minimizing the deviations $\left|\right|P_{\mu }-P_{\mu }^{\mathrm{targ}}{\left|\right|}_{F}^{2}$ and $\left|\right|P_{\sigma }-P_{\sigma }^{\mathrm{targ}}{\left|\right|}_{F}^{2}$, where $P_{\mu }^{\mathrm{targ}}$ and $P_{\sigma }^{\mathrm{targ}}$ refer to the target mean and fluctuating power distributions, and ||∙||_F_ refers to the Frobenius norm.

For demonstration, we consider a dynamic random EM field target, featuring a single circular focus area (value 1) in the normalized mean power distribution with all other regions set to 0, and distinct single focus areas in the normalized fluctuating power distribution. These configurations ensure a stable time‐invariant region, alongside a random time‐variant region. The 1‐bit RTCM consists of 16 × 16 units, each with a size of 20 mm, operating at 5 GHz. The radio source is positioned at **r**_t_ = (0, 0, 1).

**FIGURE 2:**
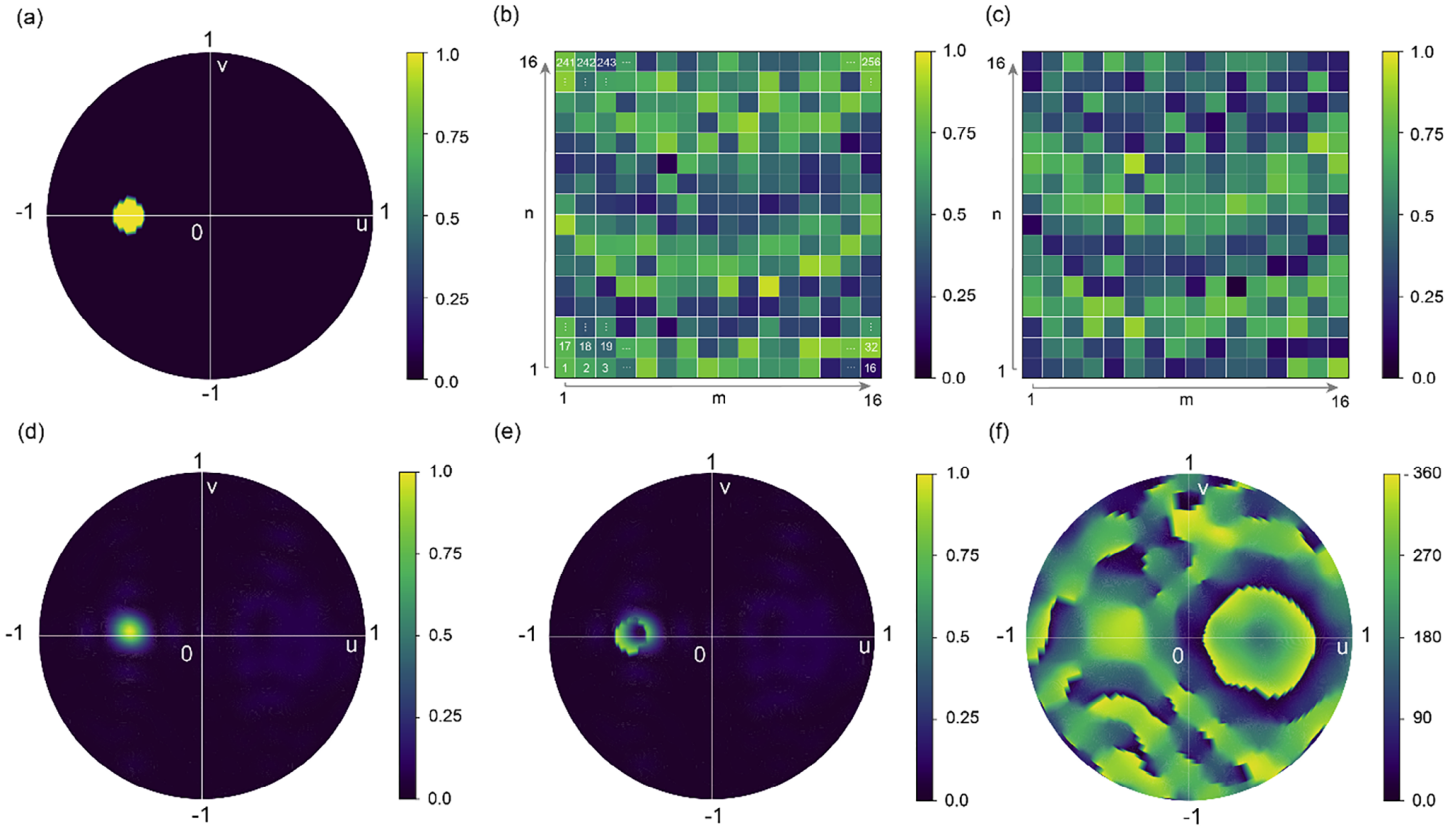
(a) The $P_{\mu }^{\mathrm{targ}}$ distribution. (b) The optimized **p**_0_. The white numbers indicate the index *l* of the units, ordered from left to right and bottom to top. (c) The optimized **p**_1_. (d) The normalized intensity of the optimized *P_μ_* distribution. (e) The error distribution of $|P_{\mu }-P_{\mu }^{\mathrm{targ}}|$. (f) The Φ*_μ_* distribution.

Figure 2a illustrates the target mean power distribution, with the far‐field region parameterized by *u* = *sinθ cosϕ* and *v* = *sinθ sinϕ*, where *θ* denotes the polar angle measured from the +*z* axis, and *ϕ* denotes the azimuthal angle measured counterclockwise from the +*x* axis in the *xy*‐plane (see Note S3 for details). The target mean power distribution features a single circular focus at (u, v) = (−0.5, 0) with a diameter of 0.1. A gradient optimization scheme is adopted to obtain the optimized marginal distributions by minimizing the loss function $L_{\mu }=\left|\right|P_{\mu }-P_{\mu }^{\mathrm{targ}}{\left|\right|}_{F}^{2}$ in accordance with Equation (2) (see Note S4 for details) [53]. Figure 2b,c shows the optimized marginal distributions **p**_0_ and **p**_1_, which exhibit complementary behavior, satisfying **p**_0_+**p**_1_ = 1. It also indicates that, unlike conventional deterministic 1‐bit coding metasurfaces, which can only realize discrete amplitude modulation of ±1, the RTCM enables continuous real‐value amplitude modulation via the marginal distribution, as also evident from Equation (2). Benefiting from this mechanism, even a 1‐bit RTCM can realize high‐resolution control of the mean distribution of the EM field in the probabilistic space.

Figure 2d illustrates that the marginal distributions successfully generate a single focus area around (u, v) = (−0.5, 0), in good agreement with the target mean power distribution. The discrepancy is primarily observed at the edge of the target pattern, as shown in Figure 2e. In addition, the mean component of the TSCF maintains a nearly uniform phase distribution around the focus region, as shown in Figure 2f. These observations suggest that the marginal distributions provide an effective means to control the mean power distribution of TSCF.

**FIGURE 3:**
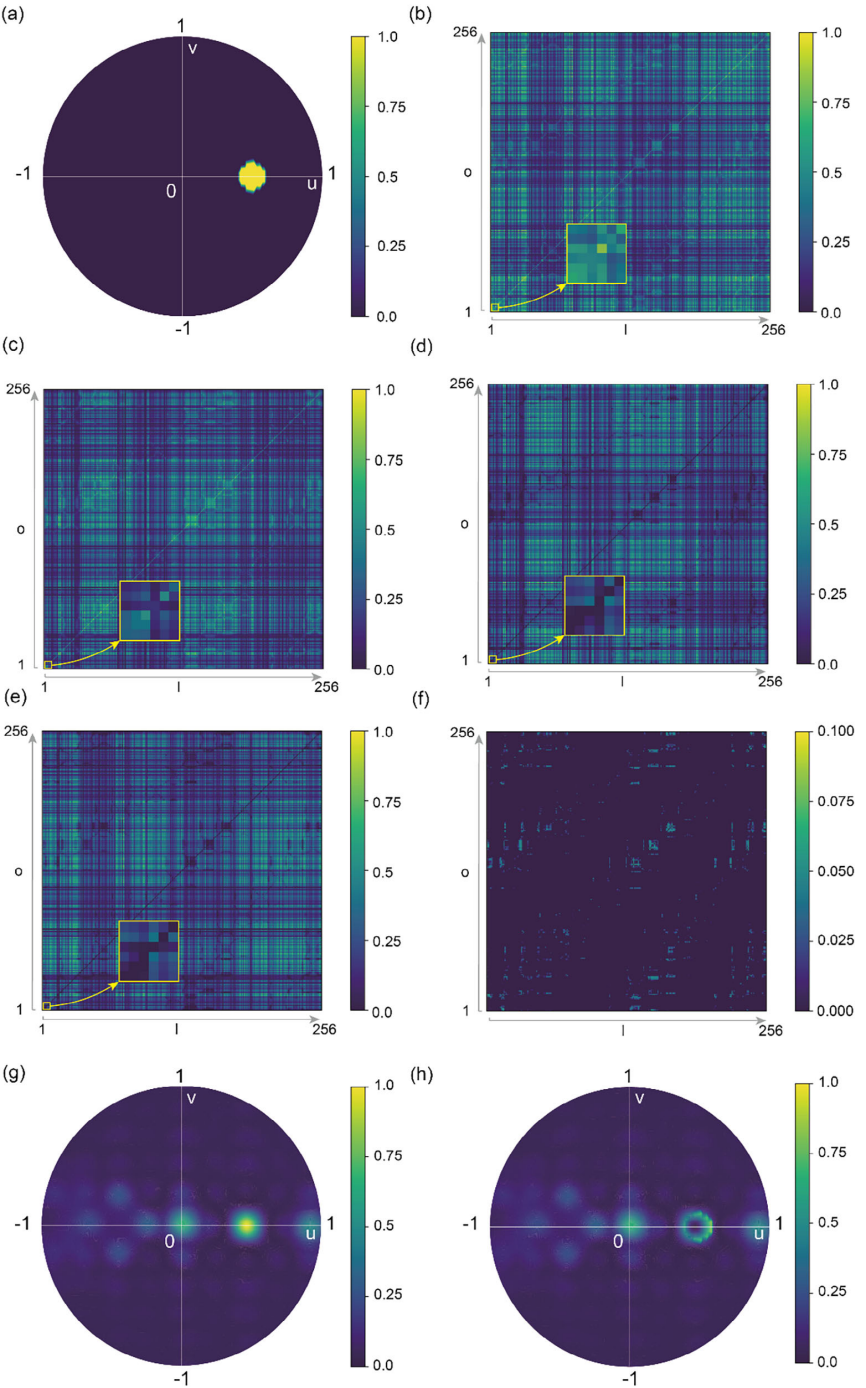
(a) The $P_{\sigma }^{\mathrm{targ}}$ distribution. (b‐e) The optimized pairwise joint distributions of **P**_00_ (b), **P**_11_ (c), **P**_01_ (d), and **P**_10_ (e). The embedded pictures refer the details of corresponding local distributions with *l* and *o* various from 2 to 7. (f) The ℒ_boundary_ of **P**_11_. (g) The normalized optimized *P_σ_* distribution. (h) The error distribution of $|P_{\sigma }-P_{\sigma }^{\mathrm{targ}}|$.

Figure 3a illustrates the target fluctuating power distribution, which features a single focus spot at (u,v) = (0.5,0) with a diameter of 0.1 (see Note S3 for details). To achieve this target, a gradient optimization scheme is adopted to obtain the optimized pairwise joint distribution according to Equation (3) (see Note S4 for details). The optimization process incorporates the constraints of **p**_1_. Specifically, **P**_11_ is constructed as **P**_11_ = **U** ^T^ **U**+**p**_1_(**p**_1_)^T^ to ensure symmetry, diagonal elements, and the positive semi‐definite condition, where **U** = **VD**. **V** is an arbitrary matrix with normalized columns, and **D** = *diag*([*d*_1_,*d*_2_,..,*d_MN_*])), with *d_l_* = (*p_l_* (1−*p_l_*))^1/2^. In addition, a boundary condition is incorporated into the loss function to guarantee the validity of **P**_11_, defined as

$$\begin{array}{c} L_{\mathrm{boundary}}=max\left\{0,p_{11}\left(l,o\right)-min\left\{p_{1}\left(l\right),p_{1}\left(o\right)\right\},\right.\\ \left.max\left\{0,p_{1}\left(l\right)+p_{1}\left(o\right)-1\right\}-p_{11}\left(l,o\right)\right\} \end{array}$$

Finally, the total loss function is expressed as $L_{\sigma }={\left(L_{boundary}\right)}^{2}+\left|\right|P_{\sigma }-P_{\sigma }^{\mathrm{targ}}{\left|\right|}_{F}^{2}$, which enforces the boundary constraints and optimizes the fluctuating power distribution.

Figure 3b–e shows the corresponding optimized pairwise joint distributions. Figure 3b,c demonstrates that **P**_00_ and **P**_11_ are symmetric, with diagonal elements equal to **p**_0_ and **p**_1_, respectively. Figure 3d,e confirms that **P**_01_ and **P**_10_ are transposes of each other with zero diagonal entries. Figure 3f illustrates the boundary loss ℒ_boundary_, where most elements are zero and the maximum boundary error is 0.053, indicating that the generated **P**_11_ largely satisfies the imposed constraints. Figure 3g shows the resulting fluctuating power distribution obtained from the optimized pairwise joint distributions, which exhibits a focused region at (u,v) = (0.5,0). Figure 3h illustrates the corresponding discrepancy relative to the fluctuating power distribution target, with the main deviations occurring at the edges of the target spot. Overall, these results confirm that the optimized fluctuating power distribution closely matches the target, demonstrating that pairwise joint distributions offer an effective approach for controlling the fluctuating power distribution.

After obtaining the marginal and pairwise joint probability distributions, the solution space of random codes is reduced from the extremely high‐dimensional space {0,1}^256^ to a lower‐dimensional subset constrained by these distributions. Sampling from this subset generates random codes that satisfy the marginal and pairwise joint probability constraints, thereby producing the EM fields with the desired spatial distributions of temporal statistical properties. The Gumbel‐Softmax process is employed to generate random coding samples [54].

**FIGURE 4:**
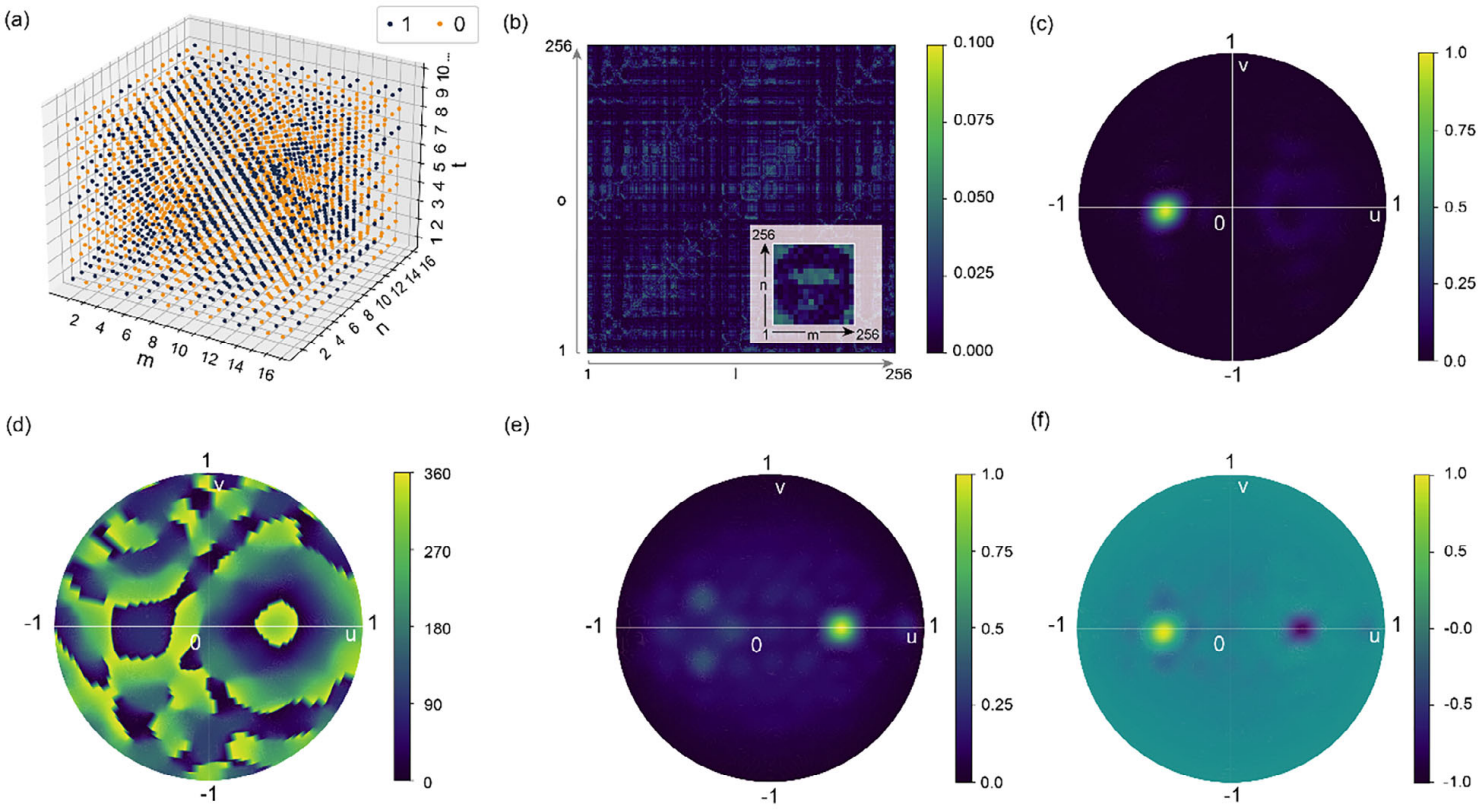
(a) The random code sequence sampled from the probability subspace constrained by the optimized margin and pairwise joint distributions. (b) The distribution of error $|P_{11}^{\mathrm{est}}-P_{11}|$, while the embedded picture demonstrates the distribution of error $|p_{1}^{\mathrm{est}}-p_{1}|$. (c) to (e) The simulated mean power distributions (c), mean phase distributions (d), and fluctuating power distribution (e) of the scattering fields. (f) The corresponding *NPD* result.

Figure 4a presents the first ten samples out of a total of 100 000 random codes. Figure 4b demonstrates the discrepancy between the estimated $P_{11}^{\mathrm{est}}$ and optimized **P**_11_, defined as $|P_{11}^{\mathrm{est}}-P_{11}|$, while the embedded picture demonstrates the discrepancy between the estimated $p_{1}^{\mathrm{est}}$ and optimized **p**_1_, defined as $|p_{1}^{\mathrm{est}}-p_{1}|$. Most element values remain close to zero, with maximum deviations of 0.051 for **P**_11_, and 0.058 for **p**_1_. These results confirm the validity of the sampled random codes.

The numerical simulation based on Equation (1) is implemented to examine the EM response of the sampled random codes through the RTCM, where the time‐varying scattering fields of the RTCM driven by the sampling random codes are calculated. Figure 4c,d presents the temporal statistical mean power and phase distribution of the simulated scattering fields, respectively. The mean power distribution exhibits a focal spot around (u,v) = (−0.5,0), while the phase distribution around the focus is uniform. Figure 4e shows the variance distribution of the simulated scattering fields, which exhibits a focus at (u,v) = (0.5,0). These results confirm that the sampling random codes can produce desired spatial control of the temporal statistical properties of EM fields.

Subsequently, we employ the normalized power difference (*NPD*), defined as *NPD* = (*P_μ_*‐*P_σ_*)/max| *P_μ_*‐*P_σ_* |, to quantify the relative intensity of the mean and fluctuating power components. Figure 4f shows the *NPD* results, with a strong positive peak with a value of 0.94 at (u,v) = (−0.5,0), indicating dominance of the mean power component, and another strong negative peak with a value of −1.0 at (u,v) = (0.5,0), indicating dominance of the fluctuating power component. These results indicate that, at the focus around (u,v) = (−0.5,0), the EM field is predominantly static over time, whereas at the focus around (u,v) = (0.5,0), the field exhibits strong temporal randomness. This distinct behavior enables undistorted signal transmission at the mean‐dominated peak and strong signal jamming at the fluctuating‐dominated peak.

## 3 Experimental Section

**FIGURE 5:**
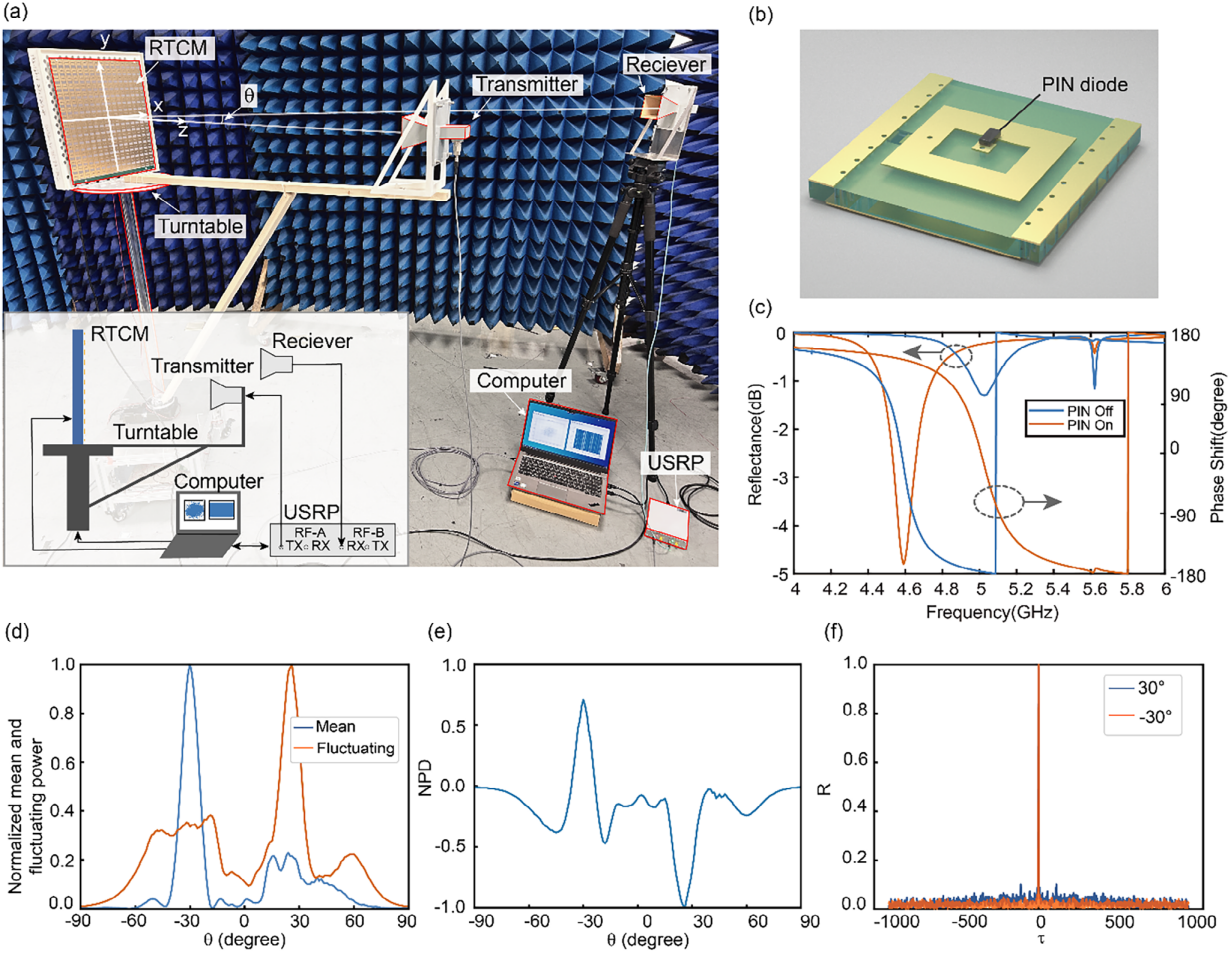
(a) The prototype of RTCM and the experiment setup of mean and fluctuating power distribution measurement. The embedded picture refers to the block diagram of experiments. (b) The unit design of the RTCM. (c) The simulation results of the reflectance and phase shift of the unit. (d) The measured mean and fluctuating power of the received signals various with *θ*. (e) the corresponding measured *NPD* of the received signals various with *θ*. (f) The autocorrelation of the received signals at −30° and 30°.

Experiments were conducted to validate the capability of the RTCM to control the spatial distribution of the temporal statistical properties of EM fields. Figure 5a shows the experimental setup and the corresponding system block diagram. The RTCM, mounted on a turntable, is controlled by a computer to play random code frames in real time. A continuous wave, emitted by the transmitting antenna, is modulated by RTCM to generate the random EM fields dynamically, which are subsequently received by the receiving antenna. The received signals are processed by a Software Defined Radio device (USRP B210) connected to a computer. The USRP device contains two independent full‐duplex RF modules (RF‐A and RF‐B). The distance from the transmitter to the RTCM is 1 m, and from the RTCM to the receiver is 2 m. The frame rate of RTCM is 50 kHz, corresponding to the switching of time‐space coding patterns 50 000 times per second.

RTCM consists of 16 × 16 unit cells, as shown in Figure 5a,b. Each unit is comprised of a PIN diode to switch between on and off states, thereby tuning its reflection property (see Note S5 for details). Figure 5c shows the simulated reflection magnitude and phase shift of the unit design, obtained using CST Studio Suite. The energy loss is relatively small, with reflectance higher than −1.3 dB. The phase shifts are approximately −180° and 0° when the PIN diode is off and on, respectively, demonstrating the capability for 1‐bit coding.

Figure 5d shows the normalized mean and fluctuating power distributions of the received signals on the *xz*‐plane with *ϕ* fixed at 0. The mean power refers to the mean intensity of the received signal, defined as ${\left|E(r(t|\theta ))\right|}^{2}$, whereas the fluctuating power corresponds to the variance of the received signal, defined as Var(*r*(*t*|*θ*)). The mean power distribution exhibits a peak at −30°, while the fluctuating power distribution peaks near 30°, in good agreement with theoretical predictions, thereby confirming the effective control of the spatial distribution of the temporal statistical characteristics. Figure 5e presents the *NPD* of the received signal. A strong positive peak at −30° indicates a stable EM region dominated by the static mean component, whereas a strong negative peak near 30° reveals a fluctuating dominated region with strong noise. The former provides a reliable channel for signal transmission, while the latter produces signal jamming. Figure 5f shows the autocorrelation of the received signals at −30° and 30°, exhibiting a Dirac‐like shape and decaying to zero as the time delay increases, confirming that the random EM process can be regarded as ergodic.

**FIGURE 6:**
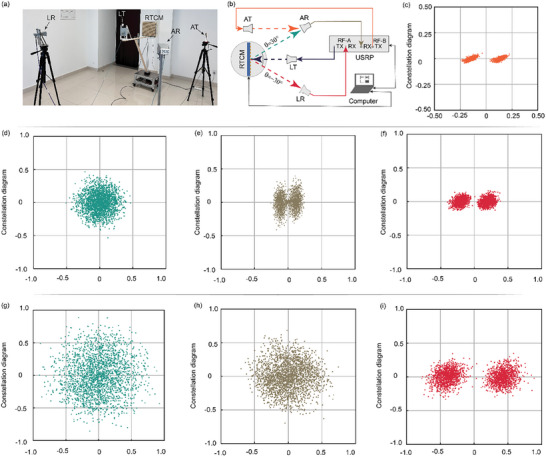
(a) Experimental setup for direct transmission and jamming. (b) Block diagram of the experiment. (c) Constellation diagram of the AR signal when AT is activated, and LT is disabled. (d–e) Transmission and interference results when LT is activated with a gain of 57 dB, and (g–i) transmission and interference results when LT is activated with a gain of 63 dB. Specifically, (d) and (g) show the constellation diagram of the AR signal when LT is activated, and AT is disabled; (e) and (h) show the AR signal when both AT and LT are activated; (f) and (i) show the LR signal when both AT and LT are activated.

Figure 6a illustrates the setup and block diagram of the radio transmission and jamming experiment. The USRP B210's RF‐A and RF‐B modules were each configured as a BPSK transmitter‐receiver pair. LR was positioned at *θ* = −30°, corresponding to the mean power peak region, to enable the radio transmission from LT to LR. AR was positioned near *θ* = 30°, corresponding to the fluctuating power peak region. Meanwhile, AT and AR form another radio link. The distance from LT to RTCM is 1m, and from RTCM to LR is 2m. The distance from AR to RTCM is 2 m, and from LT to AR is 1.73 m. The gains of LR and AR were set to 60 dB, and the gain of AT was set to 63 dB. Notably, the gain of LT was reconfigurable. Table 1 summarizes the detailed experimental configuration. When LT was disabled, and AT was activated, AR barely received the signal from AT in the absence of jamming. Conversely, when AT was disabled, and LT was activated, AR received only disturbance from RTCM. When LT and AT were activated simultaneously, LR received the direct transmission from LT, whereas AR received a superposition of AT transmission and RTCM jamming. The power of the jamming component could be tuned via the gain of LT.

**TABLE 1:** Detail configuration of the experiment setup.

LT	AT	LR	AR
Disabled	Activated	None	AT Signal
Activated	Disabled	LT Signal	Jamming
Activated (Low Gain)	Activated	LT Signal	AT Signal+ Weak Jamming
Activated (High Gain)	Activated	LT Signal	AT Signal+ Strong Jamming

First, we examine the communication performance between AT and AR by activating AT while disabling LT. Figure 6c shows the constellation diagram of the AR signal, featuring two well‐separated clusters with a BPSK amplitude of 0.137, resulting in a zero bit‐error rate (BER). Next, the weak jamming performance of RTCM was assessed by disabling AT and activating LT with a gain of 57 dB. Figure 6d shows the constellation diagram of the AR jamming signal generated via the LT‐RTCM‐AR path, which exhibits a standard deviation (STD) of 0.206, slightly larger than the BPSK amplitude. Subsequently, AT and LT were activated simultaneously to demonstrate both direct transmission and jamming. Figure 6e shows the AR received signal, which is the superposition of AT transmission and RTCM jamming. The edges of the two clusters are slightly aliased, resulting in an increased BER of 0.028. In contrast, Figure 6f shows the LR signal from LT transmitted through the stable RTCM channel, displaying two well‐separated clusters and a low BER of 0.0008.

Strong jamming performance of RTCM was further demonstrated by disabling AT and activating LT with an increased gain of 63 dB. Figure 6g shows the AR jamming signal via the LT‐RTCM‐AR path, with an STD of 0.416, substantially larger than the BPSK amplitude. When both AT and LT were activated, Figure 6h shows the AR received signal, which is fully submerged by jamming, with only a single cluster remaining a high BER of 0.4656. In contrast, Figure 6i shows the LR signal from LT transmitted through the stable RTCM channel, displaying two well‐separated clusters with higher amplitude than that in Figure 6f, maintaining a low BER of 0.0008. The detailed performance characteristics are summarized in Table 2 for comparison. These results demonstrate that RTCM can simultaneously establish a stable radio channel at θ = −30° while generating effective jamming at θ = 30°.

**TABLE 2:** Direct communication and jamming performance.

LT Gain(dB)	STD of Noise to AR	BER of AR	BER of LR
57	0.2060	0.0280	0.0008
63	0.4155	0.4656	0.0008

## 4 Discussion

This study introduces a novel scheme for controlling the spatial distribution of the temporal statistical properties of EM fields using RTCM. The proposed statistical framework reveals that the spatial distribution of both the mean and the variance of the EM fields can be manipulated via the marginal and pairwise joint probability distributions of the random codes. Numerical simulations and experimental measurements consistently validate the theoretical predictions and further demonstrate the feasibility of applying this scheme to directional transmission and jamming simultaneously.

As the array size increases, the number of elements in **P**_11_ grows quadratically, making the direct optimization computationally intractable. In such cases, the AI‐based generative approaches, such as energy‐based models (EBMs) with low‐dimensional parameterization, may provide an efficient alternative to produce random time‐space coding sequences that realize the desired EM field mean and variance distributions [55].

Compared with conventional coding metasurfaces, which mainly focus on deterministic manipulation such as beamforming or harmonic control, this work extends the control domain into the probabilistic space and establishes a mapping between the probability distributions of random codes and the spatial distribution of temporal statistical properties of EM fields. This marks a conceptual shift from deterministic pattern design to probabilistic structuring.

While the present study focuses primarily on controlling mean and variance properties of the EM field, the proposed framework is general and could be extended to more complex statistical characteristics, such as controlling higher‐order statistical moments of the EM fields. Moreover, although the framework was demonstrated using the 1‐bit coding metasurfaces for simplicity, it is not restricted to the binary implementations and can be naturally extended to multibit or continuous amplitude‐phase coding architectures, where additional modulation degrees of freedom may enrich the statistical controllability. Overall, this approach establishes statistical control as a new degree of freedom in metasurface engineering, enabling advances in communication, information security, and EM countermeasures.

## Funding

National Natural Science Foundation of China (62288101); China Postdoctoral Science Foundation (2024M760431); Independent Research Fund of the State Key Laboratory of Millimeter Waves (Z202501‐01)

## Conflicts of Interest

The authors declare no conflicts of interest.

## Supporting information




**Supporting File**: advs74826‐sup‐0001‐SuppMat.docx

## Data Availability

The data that support the findings of this study are available from the corresponding author upon reasonable request.
