# Supplementary material for: Random Time‐Space Coding Metasurfaces for Spatial Control of the Temporal Statistics of Electromagnetic Fields
Source: Adv Sci (Weinh). 2026 Mar 15;13(30):e21637. doi: 10.1002/advs.202521637 (PMC13248767; doi:10.1002/advs.202521637)
Supplement: Supplementary file 1 — Supporting File: advs74826‐sup‐0001‐SuppMat.docx [file ADVS-13-e21637-s001.docx]

Supporting Information for

Random Time-Space Coding Metasurfaces for Spatial Control of the Temporal Statistics of Electromagnetic Fields

Jia Cheng Li, Jiang Han Bao, Che Liu, and Tie Jun Cui*

**Supplementary Note 1: Modeling of random time-space coding metasurface**

The coding metasurface is composed of *M*×*N* units, each of size *d*×*d* , arranged on the *xy*-plane. The coordinate of the *l*-th unit is denoted as **r**_u_(*l*), where *l*∈{1,2,…,*MN*} is the unit index. The metasurface is assumed to be an ideal 1-bit coding state, with reflection coefficient expressed as Γ(*t*|*l*)=(−1)*^s^*(*^t^*^|^*^l^*), where the *s*(*t*|*l*)∈{0,1} represents the state function of each unit, *t* refers to time. The transmitting source is located at **r**_t_, and the receiving antenna is located at **r**_r_. The phase accumulation from source to the *l*-th unit is given by Φ_u2t_(*l*)=−*k*_0_*R*_u2t_(*l*), where *R*_u2t_(*l*) is the distance from the source to the unit, and *k*_0_ is the free-space wave vector. Similarly, the phase accumulation from the unit to the receiver is Φ_r2u_(*l*)=−*k*_0_*R*_r2u_(*l*), where *R*_r2u_(*l*) is the distance from the unit to the receiver.

A single tone signal, with amplitude of *A*_0_ and carrier frequency of *ω*_0_, is radiated from the transmitter. It is assumed that the source, receiver and metasurface are located relatively near each other such that the path delays are negligible. Accordingly, the received signal can be expressed as

$f_{r}\left(t\right)=A_{0}\exp\left(j\omega_{0}t\right)\frac{C_{0}F_{\text{u}}\left(\mathbf{r}_{r}\right)}{R_{\text{r2ms}}^{2}R_{\text{ms2t}}^{2}}\sum_{l=1}^{MN} \exp\left(j\Phi_{\text{path}}\left(l\right)\right)\Gamma\left(t|l\right)$ (S1)

where Φ_path_(*l*)=−*k*_0_(*R*_r2u_(*l*)+*R*_u2t_(*l*)) denotes the path phase accumulation, *C*_0_ is the amplitude coefficient, *F*_u_(**r**_r_) refers to the equivalent normalized radiation pattern for metasurface unit. *R*_r2ms_ and *R*_ms2t_ are the distance from the metasurface center to the receiver, and from transmitter to the metasurface center, respectively. Denoted the loss factor as *L*(**r**_t_,**r**_r_)=*C*_0_*F*_u_(**r**_r_)/(*R*_r2ms_*R*_ms2t_)^2^, Equation (S1) becomes *f*_r_(*t*|**r**_t_,**r**_r_)=*A*_0_exp(*jω*_0_*t*)*L*(**r**_t_,**r**_r_)*f*_c_(*t*|**r**_t_,**r**_r_), where *f*_c_(*t*|**r**_t_,**r**_r_) represents the time-space coding factor (TSCF), defined as

$f_{c}\left(t|\mathbf{r}_{\text{t}},\mathbf{r}_{\text{r}}\right)=\sum_{l=1}^{MN} \left(-1\right)^{s\left(t|l\right)}\exp\left(j\Phi_{\text{path}}\left(l\right)\right)$ (S2)

Once the source position and the distance between the metasurface center and the receiver are fixed, the loss factor remains nearly constant, while the path phase accumulation varies with the observation angles *θ* and *φ*, denoted as Φ_path_(*l*|*θ*,*φ*). Consequently, the TSCF becomes a function of both time and observation angles, expressed as *f*_c_(*t*|*θ*,*φ*). Here, *θ* denotes the polar angle measured from the +*z* axis, and *φ* denotes the azimuthal angle measured counterclockwise from the +*x* axis in the *xy*-plane. Moreover, the received signal is directly proportional to the TSCF, which predominantly governs its variation. Therefore, the TSCF can be used to characterize the received signal, particularly for analyzing its statistical properties.

**Supplementary Note 2: Mean and fluctuating power distribution of TSCF**

For a 1-bit random time-space coding metasurfaces (RTCM), each unit is randomly and dynamically set to either 0 or 1 during the electromagnetic (EM) manipulation process. Let the *l*-th unit have a probability *p*_1_(l)=Pr(*s*(*l*)=1) of being in the 1 state. Correspondingly, the probability of being in the 0 state is *p*_0_(l)=Pr(*s*(*l*)=0), where Pr denotes the probability operator.

The margin probabilities *p*_1_(*l*) and *p*_0_(*l*) should satisfy the general condition 0≤*p*_1/0_(*l*)≤1, and normalization condition *p*_1_(*l*)+*p*_0_(*l*)=1. Consequently, for a given marginal probability distribution, only one of *p*_1_(*l*) and *p*_0_(*l*) is independent. According to Equation (S2), the mean of TSCF is given by *μ_fc_*=−∑*_l_*(2*p*_1_(*l*)−1)exp(*j*Φ_path_(*l*)). Consequently, the mean power could be driven as

$P_{\mu}=\left|\left(2\left(\mathbf{p}_{1}\right)^{T}-\mathbf{1}^{T}\right)\exp\left(j\mathbf{\Phi}_{path}\right)\right|^{2}$ (S3)

where **p**_1_=[*p*_1_(1),*p*_1_(2),…,*p*_1_(*MN*)]^T^ denotes the probability vector of units being in the 1 state, **Φ**_path_=[Φ_path_(1), Φ _path_(2),…, Φ _path_(*MN*)]^T^ refers to the phase accumulation vector, **1**=[1,1,…,1]^T^ is the vector of ones. On the other hand, according to definition of variance, we can drive

(S4)

where *l* and *o* are unit indexes. Consequently, the mean term could be solved as

(S5)

where *p_ab_*(*l*,*o*) is the pairwise joint probability, representing the likelihood that the *l*-th unit is in state *a* and the *o*-th unit is in state *b*. Substituting Equation (S5) into Equation (S4), one can drive

$P_{\sigma}=\left\langle\left(\mathbf{P}_{00}+\mathbf{P}_{11}-\mathbf{P}_{01}-\mathbf{P}_{10}-\left(2\mathbf{p}_{1}-\mathbf{1}\right)\left(2\mathbf{p}_{1}^{T}-\mathbf{1}^{T}\right)\right)\text{,}\cos\left(\mathbf{\Phi}_{path}\mathbf{1}^{T}-\mathbf{1\Phi}_{path}^{T}\right)\right\rangle_{\text{F}}$ (S6)

where **P***_ab_*=[*p_ab_*(*l*,*o*)] denotes the pairwise joint probability function matrix, 〈*,*〉_F_ is the Frobenius inner production. Equation (S6) shows that *P_σ_* depends on both margin and pairwise joint distribution, whereas *P_μ_* depends solely on the marginal distribution. Therefore, once *P_μ_* is prescribed, the pairwise joint distribution offers an additional degree of freedom to control *P_σ_*.

The pairwise joint distribution must satisfy the general condition 0≤*p_ab_*(*l*,*o*)≤1, and the normalization condition *p*_00_(*l*,*o*)+*p*_01_(*l*,*o*)+*p*_10_(*l*,*o*)+*p*_11_(*l*,*o*)=1. Moreover, since selecting the same unit twice results in identical states, we have *p*_00/11_(*l*,*o=l*)=*p*_0/1_(*l*), *p*_01/10_(*l*, *o=l*)=0 or equivalently in matrix form *diag*(**P**_00/11_)=**p**_0/1_ and *diag*(**P**_01/10_)=**0**.

Since simultaneously switching the positions *l* and *o* along with their expected coding states *a* and *b* does not change the outcome, the pairwise joint probability is symmetric *p_ab_*(*l*,*o*)=*p_ba_*(*o*,*l*), or equivalently in matrix form **P**_01_=(**P**_10_)^T^ and **P**_00/11_=(**P**_00/11_)^T^.

According to the definitions of the marginal and pairwise joint distributions, the consistency constraint requires that *p_a_*(*l*)=*p_ab_*(*l*,*o*)+*p_aa_*(*l*,*o*), or equivalently in matrix form, **P***_ab_*+**P***_aa_*=**p***_a_***1**^T^.

By applying the above constraints, including the general normalization, diagonal element conditions, symmetry, and consistency, we can derive

$\left\{\begin{matrix} \mathbf{P}_{01}=\mathbf{1}\left(\mathbf{p}^{1}\right)^{T}-\mathbf{P}_{11} \\ \mathbf{P}_{10}=\mathbf{P}_{01}^{T} \\ \mathbf{P}_{00}=\mathbf{11}^{T}-\mathbf{p}^{1}\mathbf{1}^{T}-\mathbf{1}\left(\mathbf{p}^{1}\right)^{T}+\mathbf{P}_{11} \end{matrix}\right.$ (S7)

Equation (S7) indicates that only one of **P**_00_, **P**_01_, **P**_10_ and **P**_11_ is independent. Here, we choose **P**_11_ to characterize the remaining three pairwise joint distributions. Moreover, since all joint distributions must take values between 0 and 1, *p*_11_ should satisfy the constraint $max\{0,p_{1}\left(l\right)+p_{1}\left(o\right)-1\}\leq p_{11}\left(l,o\right)\leq min\left\{p_{1}\left(l\right),p_{1}\left(o\right)\right\}$ to ensure that all pairwise joint distributions remain valid. Substituting Equation (s7) into Equation (s6), the *P_σ_* can be finally driven as

$P_{\sigma}=4\left\langle\mathbf{P},\mathbf{C}\right\rangle_{F}$ (S8)

where **P**=**P**_11_−**p**_1_(**p**_1_)^T^, **C**=[*c*(*l*,*o*)]=[cos(Φ_path_(l)-Φ_path_(*o*))]. We have **C**=Re(exp(**Φ**_path_)exp(**Φ**_path_)^H^) , which indicates that the matrix **C** is positive semi-definite. Consequently, matrix **P** must also be positive semi-definite to guarantee that *P_σ_*≥0.

In this way, once **p**_1_ and **P**_11_ are determined, the full set of marginal and pairwise joint distributions can be obtained, and consequently, *P_μ_* and *P_σ_* can be solved. Therefore, by controlling the marginal and pairwise joint distributions, one can manipulate the mean and fluctuating power distributions of the TSCF, and thereby control the spatial distribution of the temporal statistical properties of the scattering EM fields.

**Supplementary Note 3: The synthesis process of mean and variance distribution**

For dynamically randomized scattering EM fields, energy conservation provides the physical foundation of the statistical model. Assuming the total power intercepted by the RTCM aperture is normalized to unity, the instantaneous field distribution satisfies energy conservation as

$\int_{u} \int_{v} P\left(t|u,v\right)dvdu=1$ (S9)

where *P*(*t*|*u*,*v*)=|*a*(*t*|*u*,*v*)|^2^ denotes the instantaneous power and *a*(*t*|*u*,*v*) represents the field amplitude. The field can be decomposed as *a*(*t*|*u*,*v*)= *μ*(*u*,*v*)+*δa*(*t*|*u*,*v*), where *μ*(*u*,*v*) is the time-invariant mean component and *δa*(*t*|*u*,*v*) is the zero-mean fluctuating component. Substituting this decomposition into Equation (S9) and taking the ensemble average yields

$\int_{u} \int_{v} \bar{P}\left(u,v\right)dvdu=\int_{u} \int_{v} \left(P_{\mu}\left(u,v\right)+P_{\sigma}\left(u,v\right)\right)dvdu$ (S10)

where *P_μ_*=|*μ*(*u*,*v*)|^2^ and . Equation (S10) shows that the mean and fluctuating power components jointly form the average total power distribution. Importantly, this relation indicates that the mean and variance component are not independent design targets, but originate from the same ensemble of instantaneous radiation patterns sharing a common energy budget.

**Figure S1** illustrates the constructive mechanism underlying the formation of the mean and variance envelopes through instantaneous field realizations. Figure S1a shows three representative amplitude profiles corresponding to different time instants. For the region *u*<0, all instantaneous amplitude curves exhibit an identical gate-shaped distribution located on the negative half-axis. Since the field profile remains unchanged over time in this region, averaging over different realizations preserves the same gate shape, resulting in a dominant mean component *P_μ_*. In contrast, for the region *u*>0, the three instantaneous curves exhibit different random fluctuations at each time instant. Although the detailed amplitudes vary, their fluctuations are confined within a common gate-shaped envelope. Consequently, the temporal average of the amplitude does not produce a strong deterministic mean, while the fluctuation energy accumulates to form a gate-shaped variance distribution *P_σ_*. As a result, the average power pattern is synthesized from two different physical origins: a gate-shaped distribution on the negative half-axis generated by a time-invariant mean field, and another gate-shaped distribution on the positive half-axis arising from temporal fluctuations. This example explicitly demonstrates how different instantaneous radiation patterns collectively generate prescribed mean and variance envelopes.

Therefore, the feasibility of a target pair (*P_μ_* , *P_σ_*) is determined by whether a realizable set of instantaneous amplitude distributions *a*(*t*|*u*,*v*) can be constructed, whose statistical averaging reproduces the prescribed mean and variance envelopes. In the configurations considered in this work, the mean and variance peaks are spatially separated, allowing such realizations to exist. Conversely, if no physically realizable amplitude ensemble can simultaneously support the required mean and variance envelopes, the target statistics become unattainable.

A larger aperture produces narrower radiation beams and provides additional spatial degrees of freedom for pattern synthesis. As a result, both the time-invariant mean pattern and the fluctuating variance profile can be synthesized with increased sharpness and spatial confinement, forming more localized beam clusters. The reduced beamwidth suppresses undesired angular spreading, enabling the mean and variance components to be allocated to more separated spatial directions. Consequently, the achievable spatial separation between the mean and variance patterns improves as the array size increases. In this study, the 16×16 array is used to serves as a proof-of-concept demonstration, while larger arrays are expected to provide stronger spatial separation capability and greater flexibility in shaping statistical field distributions.


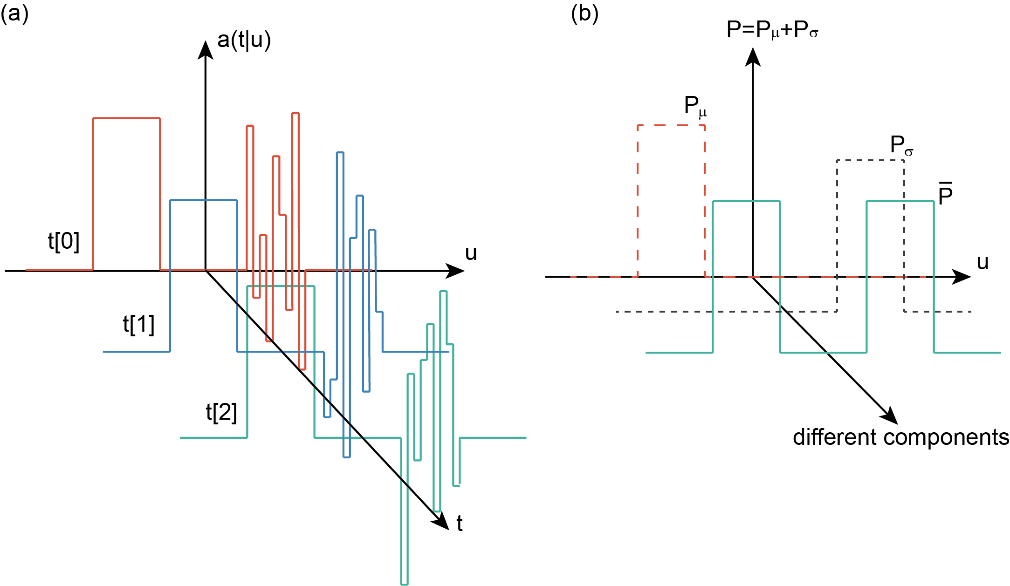


**Supplementary Figure S1.** The synthesis process of mean and variance distribution.

**Supplementary Note 4: The optimization method for p_1_ and P_11_**

The gradient-based optimization is employed to obtain **p**_1_ and **P**_11_ corresponding to the desired mean and variance distributions, respectively.

**(1) Optimization of p_1_**

The optimization workflow is illustrated in **Figure S2a**. The optimization is implemented in the Python PyTorch framework using the Adam optimizer with learning rate 0.01. The actual optimization variable is an unconstrained parameter matrix **logits** with size 16×16, matching the dimension of **p**_1_. The **logits** are initialized from a standard normal distribution. At each iteration:

1) Gradients are reset.

2) The marginal probability matrix is obtained through a differentiable mapping **p**_1_=*sigmoid*(**logits**), where *sigmoid*(*x*)=1/(1+exp(−*x*)). This parameterization guarantees 0<**p**_1_<1, thereby enforcing the probability bounds implicitly without penalty terms.

3) The mean power distribution is computed using Equation (2) in the manuscript.

4) The loss function is defined as .

Backpropagation is then performed followed by one Adam update step. The process is repeated until convergence or the maximum iteration number is reached, yielding the optimized **p**_1_.

**(2) Optimization of P_11_**

The optimization procedure for **P**_11_ follows a similar gradient-based framework (Figure S2b), also using Adam with learning rate 0.01. The optimization variable is an unconstrained matrix **logits** of size 256×256, corresponding to the joint probability matrix. Unlike **p**_1_, additional structural constraints must be satisfied, including probability bounds and positive semi-definiteness. These constraints are enforced through a differentiable parameterization rather than projection. The mapping from logits to **P**_11_ proceeds as follows:

1) Construct a column-normalized matrix **V**=|**logits**|/*colnorm*(|**logits**|), where |∙| denotes element-wise absolute value and *colnorm* represents column normalization.

2) Apply diagonal scaling **U**=**VD**, where $\mathbf{D}=diag(\sqrt{p_{1,flat}(1-p_{1,flat})})$, *diag*(∙) denotes the operator that forms a diagonal matrix from a vector and *p*_1,flat_ denotes the vectorized form of **p**_1_.

3) Construct the joint probability matrix **P**_11_=**U**^T^**U**+*p*_1,flat_ (*p*_1,flat_)^T^.

This parameterization guarantees positive semi-definiteness, consistency with the marginal probabilities, and differentiability for gradient optimization. After obtaining **P**_11_, the variance distribution is computed using Equation (3). The loss function is defined as , where

penalizes violations of admissible probability bounds. Backpropagation and optimizer updates are then performed iteratively until convergence, yielding the optimized **P**_11_.

These additional descriptions explicitly specify the optimization variables, constraint enforcement strategy, and implementation details, ensuring the correctness and reproducibility of the proposed optimization procedure. All optimization procedures are fully differentiable and reproducible using standard automatic differentiation in PyTorch.


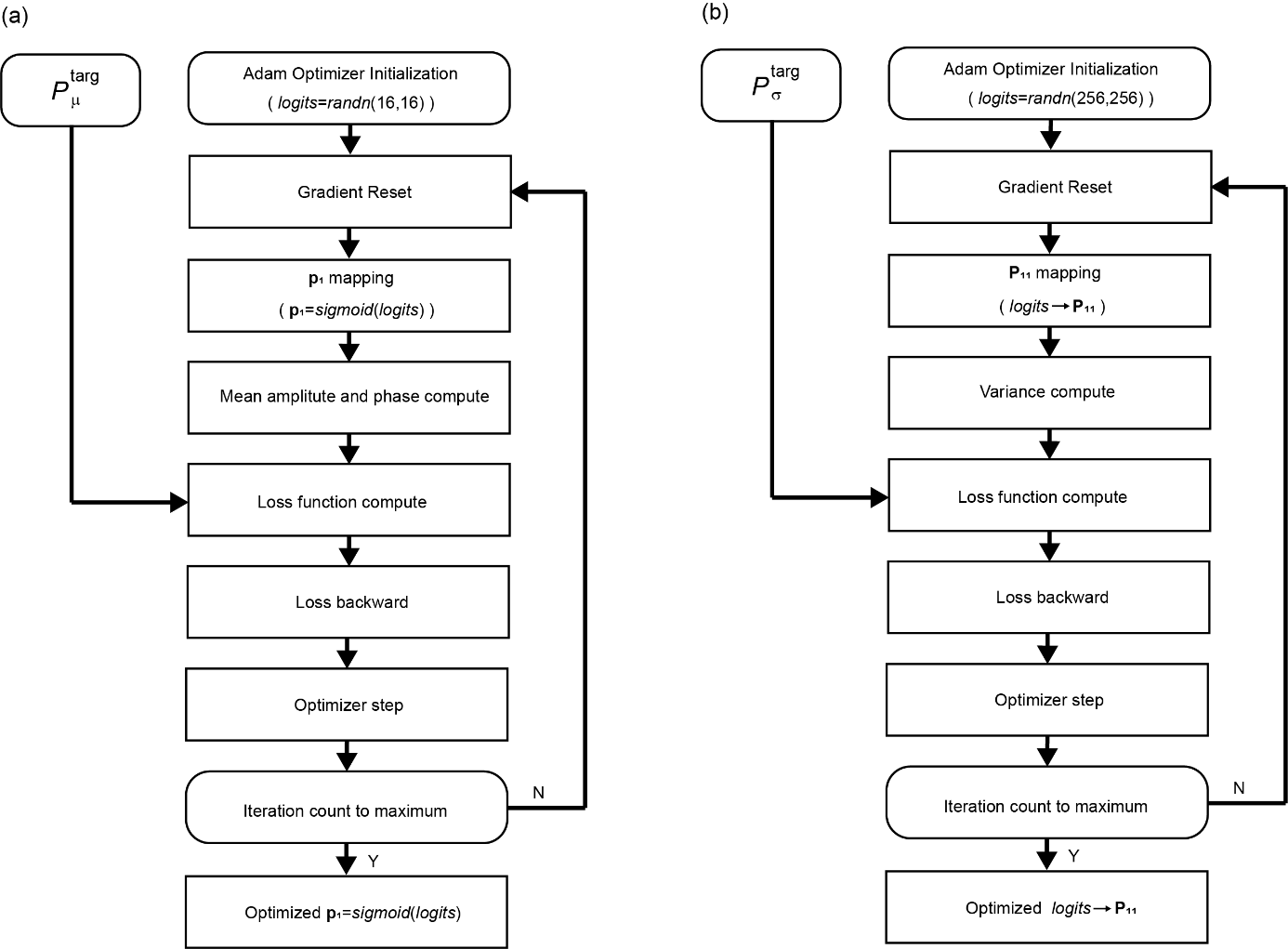


**Supplementary Figure S2.** Optimization workflows for **p**_1_ (a) and **P**_11_ (b)

**Supplementary Note 5: Metasurface design**

Supplementary **Figure S3a** and S3b shows the detailed structure of the meta-atom design, with parameters *d* = 20 mm, *a*_1_ = 14 mm, *b*_1_ = 13.65 mm, *a*_2_ = 10 mm, *b*_2_ = 2 mm, *w*_1_ = 15 mm, and *w*_2_ = 16 mm. The dielectric substrate is made of PTFE material with a thickness of 1.52 mm. The reflecting ground plane is segmented into the background on the bottom layer and wing grounds on the top layer, connected through via holes. The bias network is routed under the wing ground on the bottom layer without interfering with the radio signal. The PIN diode used is SMP1320-040LF. In our design, the S-parameter model of the PIN diode is incorporated into full-wave simulations (CST Studio) to evaluate the EM responses, including reflectance and phase shift, accounting for distribution and non-linear effects. Figure S3c shows the overall metasurface structure, and Figure S3d presents the simulated reflectance and phase shift of the meta-atom. The energy loss is small, with reflectance exceeding −1.3 dB, and the phase shifts are approximately −180° and 0° at 5 GHz when the PIN diode is off and on, respectively, demonstrating the capability for 1-bit coding.

Figure S3e and S3f demonstrate the E-field of the meta-unit with PIN on and off, respectively. The E-field is dominantly constrained between the outer and inner edge region, and the mutual coupling field is weak, ensuring the 1-bit phase modulation performance of each unit within the metasurface array. And thus, the corresponding coding sequence could be correctly implemented by setting each unit to corresponding 0 or 1 state, producing the required marginal and joint distributions.


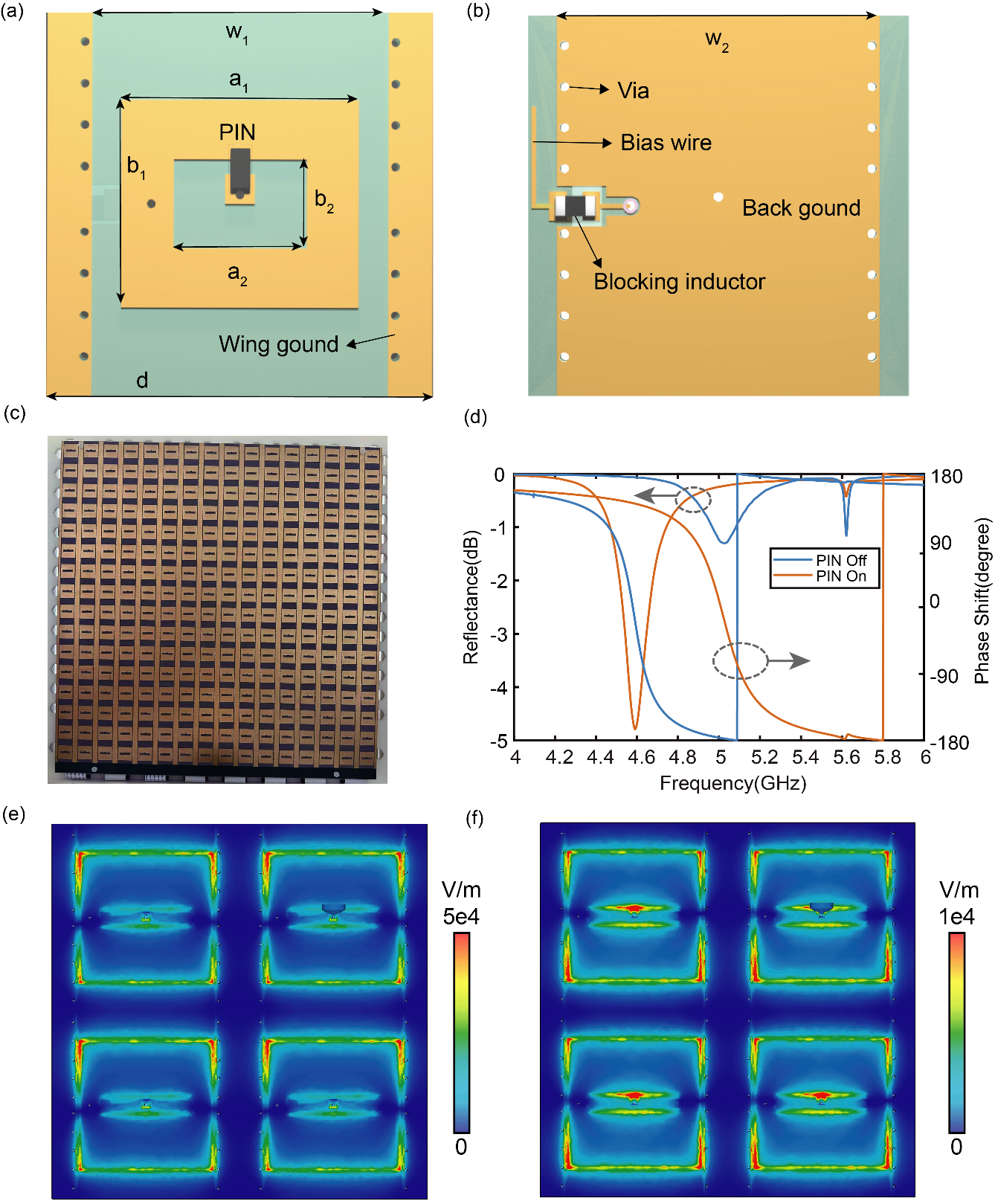


**Supplementary Figure S3.** The structure of metasurface unit. (a) Top side view of the meta-atom. (b) Bottom side view of the meta-atom. (c) The overall structure of the metasurface. (d) The reflectance and phase shift of the meta-atom. (e) and (f) The E-field distribution of PIN on and off respectively.

**Supplementary Note 6: The model of the adversarial received signal**

The received signal at the adversarial receiver can be expressed as

*r*_AR_[*k*] = *s*_AT_[*k*]+*n*_RTCM_[*k*]

where *s*_AT_[*k*] denotes the received BPSK symbol and *n*_RTCM_[*k*] represents the stochastic EM field generated by the RTCM.

Even if the adversary is able to estimate the statistical properties of *n*_RTCM_[*k*], such as its variance, this knowledge does not provide access to its instantaneous realization at each symbol time. The stochastic process produced by the RTCM is non-repetitive and physically generated in real time, and therefore cannot be deterministically predicted or reconstructed.

For small variance levels, the interference induced by *n*_RTCM_[*k*] does not significantly shift the received signals across the decision boundary, and reliable symbol detection remains possible. As the variance of *n*_RTCM_[*k*] increases to a level comparable to the signal amplitude, a portion of the received signals are displaced beyond the decision threshold, leading to symbol errors that cannot be identified or corrected without knowledge of the instantaneous noise values. When the variance becomes significantly larger than the signal amplitude, the received signals are dominated by the stochastic field, and the decision outcome is primarily determined by *n*_RTCM_[*k*], making reliable recovery infeasible.

Therefore, within this additive model, successful inversion would require complete knowledge of the instantaneous value of *n*_RTCM_[*k*] at every symbol time. Since the RTCM generates non-repeating stochastic realizations that are not observable by the adversary, partial or adaptive cancellation is not achievable even if the statistical design methodology is known.

In our security analysis, both the legitimate and adversarial transmitters are assumed to operate at the same carrier frequency and symbol rate under single carrier BPSK modulation. Under this configuration, the adversary can readily estimate the statistical characteristics of the stochastic field generated by the RTCM, such as its variance, from received signals. However, knowledge of statistical parameters does not provide access to the instantaneous realization of the stochastic field at each symbol time, and thus, does not enable deterministic cancellation.
